# Which Surgeries Are the Best Choice for Chronic Pancreatitis: A Network Meta-Analysis of Randomized Controlled Trials

**DOI:** 10.3389/fsurg.2021.798867

**Published:** 2022-02-03

**Authors:** Yu Mou, Yi Song, Hong-Yu Chen, Xing Wang, Wei Huang, Xu-Bao Liu, Neng-Wen Ke

**Affiliations:** ^1^Department of Pancreatic Surgery, West China Hospital, Sichuan University, Chengdu, China; ^2^Geriatrics Center, West China Hospital, Sichuan University, Chengdu, China; ^3^Department of Integrated Traditional Chinese and Western Medicine, West China Hospital, Sichuan University, Chengdu, China

**Keywords:** chronic pancreatitis, pancreatectomy, abdominal pain, exocrine pancreatic insufficiency, network meta-analysis

## Abstract

**Background:**

Surgery is an effective choice for the treatment of chronic pancreatitis (CP). However, there is no clear consensus regarding the best choice among the surgical procedures. The aim of this study is to conduct a network meta-analysis of randomized controlled trials comparing treatment outcomes to provide high-quality evidences regarding which is the best surgery for CP.

**Methods:**

A systematic search of the PubMed (MEDLINE), SCIE, EMBASE, CENTRAL, and CDSR databases were performed to identify studies comparing surgeries for CP from the beginning of the databases to May 2020. Pain relief and mortality were the primary outcomes of interest.

**Results:**

Ten studies including a total of 680 patients were identified for inclusion. PPPD had a better postoperative short-term pain relief and quality of life (QOL), but a worse pancreatic exocrine function deficiency and high morbidity. Berne had a significant postoperative long-term pain relief and mortality with a lower risk of pancreatic exocrine function deficiency.

**Conclusion:**

The main surgical procedures including the PPPD, Beger procedure, Frey modification and Berne modification can efficaciously treat CP. The Berne modification may be first choice with better efficacy and less complications in pancreatic function, but the impact of postoperative QOL cannot be ignored. Furthermore, when the CP patients have a mass in the pancreatic head which cannot be distinguished from pancreatic cancer, the only legitimate choice should be PPPD or classical pancreaticoduodenectomy.

## Background

Chronic pancreatitis (CP) is a progressive and irreversible fibroinflammatory disorder which is characterized by continuous destruction of the pancreatic parenchyma and fibrosis, leading to the intractable pain and poor quality of life (QOL). As the disease progresses, there is consecutive loss of pancreatic function and development of local complications (e.g., ductal obstruction, pancreatic pseudocysts, etc.) (1, 2). The risk of pancreatic cancer is increased in patients with CP (1). These recurrent or persistent pain and comorbidities make CP one of the most resource-consuming diseases (3, 4).

Since the tormenting pain is the predominant symptom of CP, the primary goal of the treatment is to mitigate the pain (1, 5–7). However, the exact mechanisms of abdominal pain in CP have not been fully elucidated. Therefore, adequate management of pain in CP remains a challenge (5). Traditionally, a conservative step-up approach has been advocated for pain treatment in CP which consists of medical, endoscopic, and surgical therapy (1, 6, 7). Research has proved a large proportion of patients refractory to medical therapy (8, 9). Endoscopic therapy is indicated in such patients when there is evidence of biliary or pancreatic ductal obstruction and symptomatic pseudocysts (1, 6, 7). However, it has been shown in several randomized controlled trials (RCTs) (10–12) that endoscopic therapy is not as efficacious as surgery (either resection or drainage) in terms of pain relief. Cahen et al. (11, 12) reported that more than half of the patients who were initially randomized to endoscopic therapy eventually underwent surgery for pain control.

Therefore, increasing evidence supports that surgery, even early surgery (13–15), is an optional choice for the treatment of CP. However, there are several surgical procedures for CP, such as pylorus-preserving pancreaticoduodenectomy (PPPD), Beger procedure, Frey modification and so on. There is no clear consensus regarding the best choice among the surgical procedures. Several systematic reviews have tried to analyze the difference among surgical procedures for CP (14, 16–20); however, most of them included retrospective studies or only compared two surgical procedures. In the present study, we compared all surgical procedures for CP and each included study was RCT. The aim of this study was to conduct a meta-analysis of RCTs comparing treatment outcomes and operation time to provide high-quality evidence regarding which is the best surgical technique for CP.

## Methods

### Literature Search

Electronic databases such as PubMed (Medline), Science Citation Index Expanded, EMBASE, Cochrane Central Register of Controlled Trials and the Cochrane Database of Systematic Reviews were searched for studies comparing surgical techniques for CP from the beginning of databases to May 2020. The following medical subject headings (MeSH) were used: “pancreaticoduodenectomy,” “pancreatoduodenectomy,” “Whipple,” “pancreatoduodenal resection,” “pylorus preserving pancreaticoduodenectomy,” “PPPD,” “Pancreaticojejunostomy,” “Beger,” “Frey” “Puestow or Partington-Rochelle,” “pancreatic surgery,” “randomized controlled trial,” and “controlled clinical trial”; combinations of these headings were used for word searches. In the PubMed database, we used the following strategy: (pancreaticoduodenectomy OR pancreatoduodenectomy OR “Whipple procedure” OR “pancreatoduodenal resection” OR PPPD OR Pancreaticojejunostomy OR “Beger procedure” OR “Frey procedure” OR “Frey modification” OR “Puestow procedure” OR “Partington-Rochelle”) AND (“randomized controlled trial” OR “controlled clinical trial”). We performed a cross-reference search of all selected articles in case studies were missed during the initial database searches. The inclusion of articles was determined by the consensus of two authors; when this failed, a third author adjudicated.

### Inclusion and Exclusion Criteria

All studies were scrutinized for eligibility by two authors using the following inclusion criteria: (1) studies described RCTs. Studies written in languages other than English were translated by a native speaker of that language who also holds a medical degree and with familiarity in surgery or in gastroenterology. (2) Human clinical trials compared surgical procedures for CP. (3) The full-text articles were published in peer-reviewed journals.

Studies were excluded if the following conditions existed: (1) they were case reports, reviews, letters, editorials or expert opinions; (2) the primary outcomes were unavailable; (3) they included surgery for indications other than CP including hepatopancreatobiliary malignancy, pancreas divisum, pancreatic hemorrhage or pancreatic infection, or they were studies pertaining only to drainage of cysts or pseudocysts and pancreatic transplant; and (4) they were trials comparing endoscopic vs. surgical procedures, or a surgical procedure vs. conservative treatment for CP.

### Outcomes of Interest

Pain relief and mortality were the primary outcomes of interest. Secondary outcomes were QOL, new-onset endocrine and exocrine pancreatic insufficiency, and postoperation complications (including fistulas, postoperative hemorrhage, intra-abdominal abscesses, anastomotic leakage, sepsis and wound infections).

### Data Extraction

Data were extracted by two independent authors using standardized proformas and included participant characteristics, study characteristics, data needed for the methodological quality assessment of the study, and primary and secondary outcomes, according to availability. Data regarding participant characteristics included number of participants in each group, age, sex, etiology, etc. Data regarding study characteristics included study design, sample size information, follow-up period, loss to follow-up, and surgical procedures compared. Means of the outcomes were used for meta-analytical synthesis by default. If medians rather than means were available in some studies, means were estimated as the medians when the samples were greater than 25. With samples of any size smaller than 25, the means were estimated using the following formula: (low end of range + median^*^2 + high end of range)/4. The standard deviations were estimated as range/4 when only a range was given (21). The risk of bias of all selected studies was assessed using the Cochrane Collaboration's tool for assessing risk of bias for RCTs.

### Statistical Analysis

The statistical analysis was performed using software R 4.0.3 (main packages include gemtc and rjags). A network of all surgical approaches was mapped. Contribution plots were performed to display contributions of each direct comparison in network meta-analysis. Continuous variables were estimated as weighted mean difference (WMD) with their corresponding 95% confidence interval (CI), while categorical variables were expressed as odds ratio (OR) with 95% CI. Heterogeneity was assessed using Chi-square tests and a *P* < 0.1 was considered significant. *I*^2^ statistic were used for the evaluation of statistical heterogeneity: an *I*^2^ value of 50% or more was indicative of the presence of heterogeneity (22). The fixed effects models were initially applied (23), while the random effects model was used if the assumption of homogeneity of studies was rejected (24). A *P* < 0.05 was considered significant in the meta-analytical synthesis. Descriptive methods were utilized if the data were considered to be inappropriate for meta-analytical synthesis.

### Ethics Statement

The data used in this meta-analysis was derived from articles published in peer-reviewed journals. This study does no harm to the subjects included in the study, so ethical approval and consent to participate is not necessary for this study.

## Results

### Study Selection

**Figure 1:**
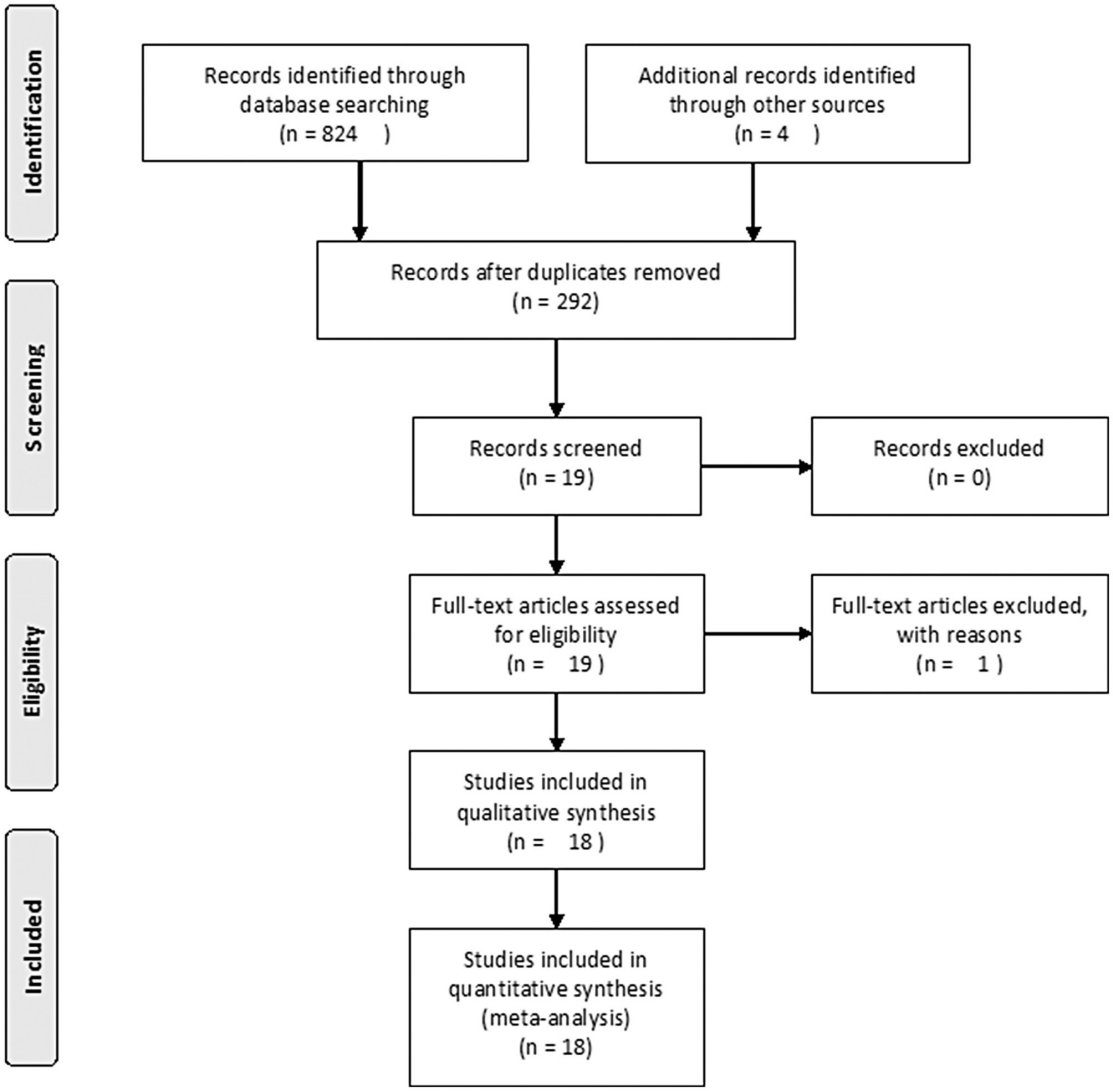
PRISMA flow diagram.

The search strategy initially identified 292 relevant studies. Nineteen publications (25–43) with full-text were identified for detailed investigation after filtering the studies. Of these, two trials were described in eight publications (25–32), and two of the trials with long-term follow-up were described in four publications (33, 34, 36, 37). One study (41) had no data available. Finally, 11 studies were identified for inclusion with a total of 680 patients (Figure 1). Table 1 outlines the key design features of each study. Table 2 shows the characteristics of each study. One study compared PPPD with the Frey modification. Two studies compared the Beger procedure with the Frey modification. One study compared the Beger procedure with the Berne modification. Four studies compared the Beger procedure with PPPD. Two studies compared the Berne modification with PPPD. The differences and similarities of the operations for CP are shown in Table 3. The Cochrane Collaboration tool for assessing risk of bias (Table 4) showed that all studies had no domain with a high risk of bias, and 5 studies had no information about how randomization was performed.

**Table 1:** Characteristics of included studies.

	**Study**	**Year**	**Country**	**Study design**	**Study period**	**Comparison**	**Total patients included**	**Patients followed up, *n***	**Loss to follow up**	**Median follow-up time**
1	J. R. Izbicki	1998	Germany	RCT	1995–1997	PPPD vs. Frey	61			
1.1	J. R. Izbicki and C. E. Broelsch	1998	Germany	RCT	1995–1997	PPPD vs. Frey	61	61	0	2 yr
1.2	Tim Strate and J. R. Izbicki et al.	2008	Germany	RCT	1995–1997	PPPD vs. Frey	61	58	3	7 yr
1.3	K. Bachmann and J. R. Izbicki et al.	2013	Germany	RCT	1995–1997	PPPD vs. Frey	61	60	1	15 yr
2	J. R. Izbicki	1995	Germany	RCT	1992–1994	Beger vs. Frey	74			
2.1	J. R. Izbicki and C. E. Broelsch	1995	Germany	RCT	1992–1994	Beger vs. Frey	74	42	0	mean 1.5 yr
2.2	J. R. Izbicki and C. E. Broelsch	1997	Germany	RCT	1992–1994	Beger vs. Frey	74	74	0	30 mo
2.3	C. Bloechle and J. R. Izbicki	1997	Germany	RCT	1992–1994	Beger vs. Frey	74	30	0	30 mo
2.4	Tim Strate and J. R. Izbicki et al.	2005	Germany	RCT	1992–1994	Beger vs. Frey	74	67	7	8.5 yr
2.5	K. Bachmann and O. Mann	2014	Germany	RCT	1992–1994	Beger vs. Frey	74	71	3	16 yr
3	Marcus W. Buchler	1995	Germany	RCT	1991–1993	Beger vs. PPPD	40			
3.1	Marcus W. Buchler and Hans G. Beger	1995	Germany	RCT	1991–1993	Beger vs. PPPD	40	31	9	6 mo and 10 d
3.2	M. W. Muller and M. W. Buchler	2008	Germany	RCT	1991–1993	Beger vs. PPPD	40	27 (7 yr follow-up)	13	7 yr
								29 (14 yr follow-up)	11	14 yr
4	Michael W. Muller and Markus W. Buchler	1997	Switzerland	RCT	N/A	Beger vs. PPPD	20	20	0	PPPD 26 mo
										Beger 24 mo
5	Markus W. Büchler	2008	Germany	RCT	2002–2005	Beger vs. Berne	65			
5.1	Jorg Koninger and Markus W. Buchler	2008	Germany	RCT	2002–2005	Beger vs. Berne	65	58	7	2 yr
5.2	Ulla Klaiber and Markus K. Diener	2016	Germany	RCT	2002–2005	Beger vs. Berne	65	51	14	129 mo
6	Tobias Keck and Ulrich T. Hopt	2012	Germany	RCT	1997–2001	Beger vs. PPPD	87	85	2	65.6 mo
7	Gyula Farkas and Gyula Farkas Jr.	2006	Hungary	RCT	2002–2004	Berne (OPPHR) vs. PPPD	40	40	0	1 yr
8	I. Klempa and W. Arnold	1995	Germany	RCT	1987–1993	Beger vs. PPPD	43	43	0	36–66 mo
9	Tsann-Long Hwang	2001	China Taiwan	RCT	1998–2001	DP vs. PD	18	18	0	6–36 mo
10	J. R. Izbicki and C. E. Broelsch	1995	Germany	RCT	N/A	Beger vs. Frey	26	24	2	mean 12 mo
11	M. K. Diener	2017	Europe	RCT	2009–2013	DPPHR (mainly Berne) vs. PPPD	250	226	24	24 mo

*RCT, randomized controlled trial; PPPD, pylorus-preserving pancreaticoduodenectomy; DP, distal pancreatectomy; PD, pancreaticoduodenectomy; OPPHR, organ-preserving pancreatic head resection; DPPHR, duodenum-preserving pancreatic head resection; N/A, not applicable; yr, year(s); mo, month(s); d, day(s)*.

**Table 2:** Baseline patient demographics for all included studies.

	**Study**	**Gender**		**Etiology**	**Age**	**Time since onset of symptoms**	**Pancreatic head enlargement**	**Cambridge classification**
		**Male**	**Female**					
1.1	J. R. Izbicki and C. E. Broelsch	51	10	AL (47) ID (14)	PPPD 44.6 ± 5.3	PPPD 4.8 ± 2.6 yr	>35 mm (mean 56 mm)	Stage I 0, stage II 14, stage III 42
					Frey 43.1 ± 6.5	Frey 5.5 ± 2.3 yr		
1.2	Tim Strate and J. R. Izbicki et al.	51	10	AL (47) ID (14)	PPPD 44.6 ± 5.3	PPPD 4.8 ± 2.6 yr	>35 mm (mean 56 mm)	Stage I 0, stage II 14, stage III 44
					Frey 43.1 ± 6.5	Frey 5.5 ± 2.3 yr		
1.3	K. Bachmann and J. R. Izbicki et al.	51	10	AL (47) ID (14)	PPPD 44.6 ± 5.3	PPPD 4.8 ± 2.6 yr	>35 mm (mean 56 mm)	Stage I 0, stage II 14, stage III 46
					Frey 43.1 ± 6.5	Frey 5.5 ± 2.3 yr		
2.1	J. R. Izbicki and C. E. Broelsch	54	20	AL (51) ID (21) TR (1) IA (1)	Beger 43.5 ± 7.2	Beger 5.3 ± 2.4 yr	>35 mm	N/A
					Frey 42.2 ± 6.4	Frey 4.8 ± 2.7 yr		
2.2	Izbicki, J. R. and C. E. Broelsch et al.	31	11	AL (30) ID (10) TR (1) IA (1)	Beger 45.3 ± 8.1	Beger 5.9 ± 2.5 yr	>35 mm	Stage I 4, stage II 12, stage III 19
					Frey 44.1 ± 5.9	Frey 6.4 ± 2.8 yr		
2.3	C. Bloechle and J. R. Izbicki	22	8	AL (24) ID (6)	Beger 44.4 ± 6.6 ^†^	Beger 6.1 ± 2.6 yr ^†^	>35 mm	N/A
					Frey 45.6 ± 5.4 ^†^	Frey 5.5 ± 2.8 yr ^†^		
2.4	Tim Strate and J. R. Izbicki et al.	54	20	AL (51) ID (21) TR (1) IA (1)	Beger 43.5 ± 7.2	Beger 5.3 ± 2.4 yr	>35 mm	N/A
					Frey 42.2 ± 6.4	Frey 4.8 ± 2.7 yr		
2.5	K. Bachmann and O. Mann	54	20	AL (51) ID (21) TR (1) IA (1)	Beger 43.5 ± 7.2	Beger 5.3 ± 2.4 yr	>35 mm	N/A
					Frey 42.2 ± 6.4	Frey 4.8 ± 2.7 yr		
3.1	Marcus W. Buchler and Hans G. Beger	36	4	AL (34) OT (6)	PPPD 46 ± 11	PPPD 62 ± 71 mo	>40 mm	N/A
					Beger 43 ± 9	Beger 61 ± 55 mo		
3.2	M. W. Muller and M. W. Buchler	36	4	AL (34) OT (6)	PPPD 46 ± 12	PPPD 62 ± 71 mo	>40 mm	N/A
					Beger 43 ± 10	Beger 61 ± 56 mo		
4	Michael W. Muller and Markus W. Buchler	19	1	AL (15) OT (5)	PPPD 44.5	N/A	Enlarged^‡^	N/A
					Beger 45.5			
5.1	Jorg Koninger and Markus W. Buchler	45	20	N/A	Beger 48 ± 12	Beger 3 (0.2–20) yr	N/A	N/A
					Berne 46 ± 11	Berne 3.6 (0.1–20) yr		
5.2	Ulla Klaiber and Markus K. Diener	45	20	N/A	Beger 48 ± 12	N/A	N/A	N/A
					Berne 46 ± 11			
6	Tobias Keck and Ulrich T. Hopt	72	13	AL (73) ID (12)	PPPD 42.7	PPPD 36 mo	N/A	N/A
					Beger 41.2	Beger 60 mo		
7	Gyula Farkas and Gyula Farkas Jr.	30	10	N/A	PPPD 45 ± 8	PPPD 7 ± 9 yr	>40 mm	N/A
					OPPHR 43 ± 5	OPPHR 8 ± 4 yr		
8	I. Klempa and W. Arnold	33	10	AL (33) ID (10)	PPPD 47	PPPD 5.7 yr	N/A	N/A
					Beger 46	Beger 6.8 yr		
9	Tsann-Long Hwang	16	2	N/A	DP 46.2 ± 8.5	DP 2.4 ± 0.7 yr	N/A	N/A
					PD 52.38 ± 11.8	PD 1.5 ± 0.9 yr		
10	J. R. Izbicki and C. E. Broelsch	19	7	AL (21) IA (1) ID (4)	Beger 46.8	Beger 5.9 yr	>35 mm	Stage I 2, stage II 6, stage III 18
					Frey 41.7	Frey 6.6 yr		
11	M. K. Diener	181	45	N/A	DPPHR 52.3 ± 11.1	DPPHR 42.4 ± 89.1 mo	Enlarged^‡^	N/A
					PPPD 51.5 ± 10.5	PPPD 38.1 ± 48.6 m		

*PPPD, pylorus-preserving pancreaticoduodenectomy; DP, distal pancreatectomy; PD, pancreaticoduodenectomy; OPPHR, organ-preserving pancreatic head resection; DPPHR, duodenum-preserving pancreatic head resection; N/A, not applicable; AL, alcoholic; ID, idiopathic; TR, traumatic; IA, iatrogenic; OT, other; yr, year(s); mo, month(s)*;

† *Combined with two groups*;

‡ *with enlargement but size not mentioned*.

**Table 3:** Differences and similarities of the operations for CP.

	**Extent of resection**	**Ductal decompression**	**Methods of anastomisis**
PPPD	Total pancreatic head, partial duodenal, common bile duct	Transection	End-to-side pancreaticojejunostomy, choledochojejunostomy and jejunoduodenostomy
Beger procedure	Almost all of the pancreatic head	Transection	End-to-side pancreaticojejunostomy
Berne modification	Partial pancreatic head	Wide opening in the pancreatic head	End-to-side pancreaticojejunostomy
Frey modification	Partial pancreatic head	Longitudinal opening the pancreatic duct	Side-to-side pancreaticojejunostomy

**Table 4:** The Cochrane Collaboration's tool for assessing risk of bias of RCTs.

	**Study**	**Random sequence generation**	**Allocation concealment**	**Blinding of participants**	**Blinding of outcome assessment**	**Incomplete outcome data**	**Selective reporting**	**Other bias**
1.1	J. R. Izbicki and C. E. Broelsch	Low risk	Low risk	Low risk	Low risk	Low risk	Low risk	Low risk
1.2	Tim Strate and J. R. Izbicki et al.	Low risk	Low risk	Low risk	Low risk	Low risk	Low risk	Low risk
1.3	K. Bachmann and J. R. Izbicki et al.	Low risk	Low risk	Low risk	Low risk	Low risk	Low risk	Low risk
2.1	J. R. Izbicki and C. E. Broelsch	Low risk	Low risk	Low risk	Low risk	Low risk	Low risk	Low risk
2.2	J. R. Izbicki and C. E. Broelsch et al.	Low risk	Low risk	Low risk	Low risk	Low risk	Low risk	Low risk
2.3	C. Bloechle and J. R. Izbicki	Low risk	Low risk	Low risk	Low risk	Low risk	Low risk	Low risk
2.4	Tim Strate and J. R. Izbicki et al.	Low risk	Low risk	Low risk	Low risk	Low risk	Low risk	Low risk
2.5	K. Bachmann and O. Mann	Low risk	Low risk	Low risk	Low risk	Low risk	Low risk	Low risk
3.1	Marcus W. Buchler and Hans G. Beger	Unclear	Unclear	Low risk	Low risk	Low risk	Low risk	Low risk
3.2	M. W. Muller and M. W. Buchler	Unclear	Unclear	Low risk	Low risk	Low risk	Low risk	Low risk
4	Michael W. Muller and Markus W. Buchler	Unclear	Unclear	Low risk	Low risk	Low risk	Low risk	Low risk
5.1	Jorg Koninger and Markus W. Buchler	Low risk	Low risk	Low risk	Low risk	Low risk	Low risk	Low risk
5.2	Ulla Klaiber and Markus K. Diener	Low risk	Low risk	Low risk	Low risk	Low risk	Low risk	Low risk
6	Tobias Keck and Ulrich T. Hopt	Unclear	Unclear	Low risk	Low risk	Low risk	Low risk	Low risk
7	Gyula Farkas and Gyula Farkas Jr.	Unclear	Unclear	Low risk	Low risk	Low risk	Low risk	Low risk
8	I. Klempa and W. Arnold	Low risk	Low risk	Low risk	Low risk	Low risk	Low risk	Low risk
9	Tsann-Long Hwang	Low risk	Low risk	Low risk	Low risk	Low risk	Low risk	Low risk
10	J. R. Izbicki and C. E. Broelsch	Low risk	Low risk	Low risk	Low risk	Low risk	Low risk	Low risk
11	M. K. Diener	Low risk	Low risk	Low risk	Low risk	Low risk	Low risk	Low risk

### Postoperative Pain Relief

**Figure 2:**
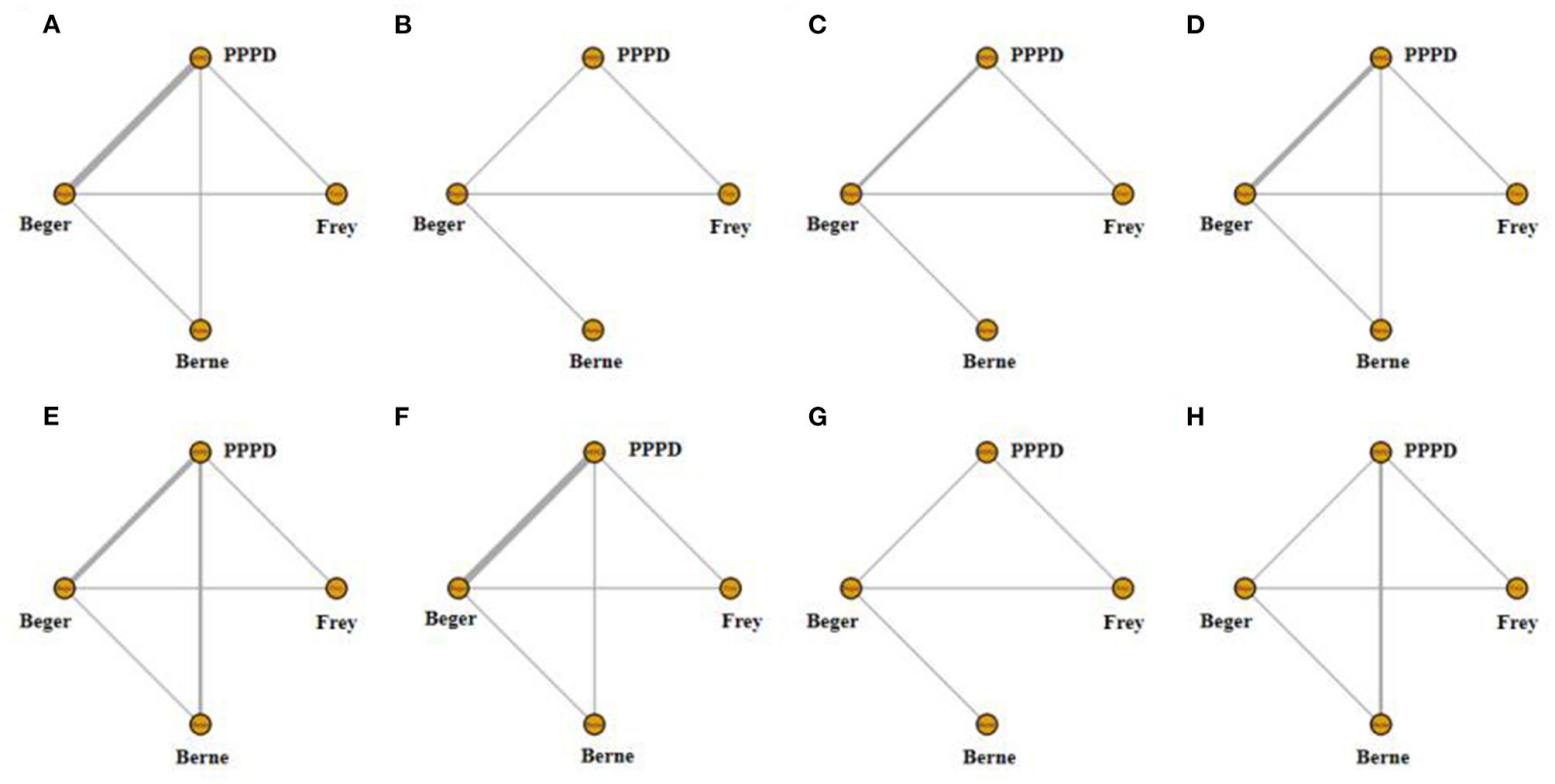
Network of treatment comparisons. The width of the lines inflects the number of trials comparing each pair of treatments. **(A)** Network of short-term pain relief; **(B)** Network of long-term pain relief; **(C)** Network of quality of life; **(D)** Network of pancreatic exocrine function deficiency; **(E)** Network of pancreatic endocrine function deficiency; **(F)** Network of morbidity; **(G)** Network of long-term mortality; **(H)** Network of operation time.

**Figure 3:**
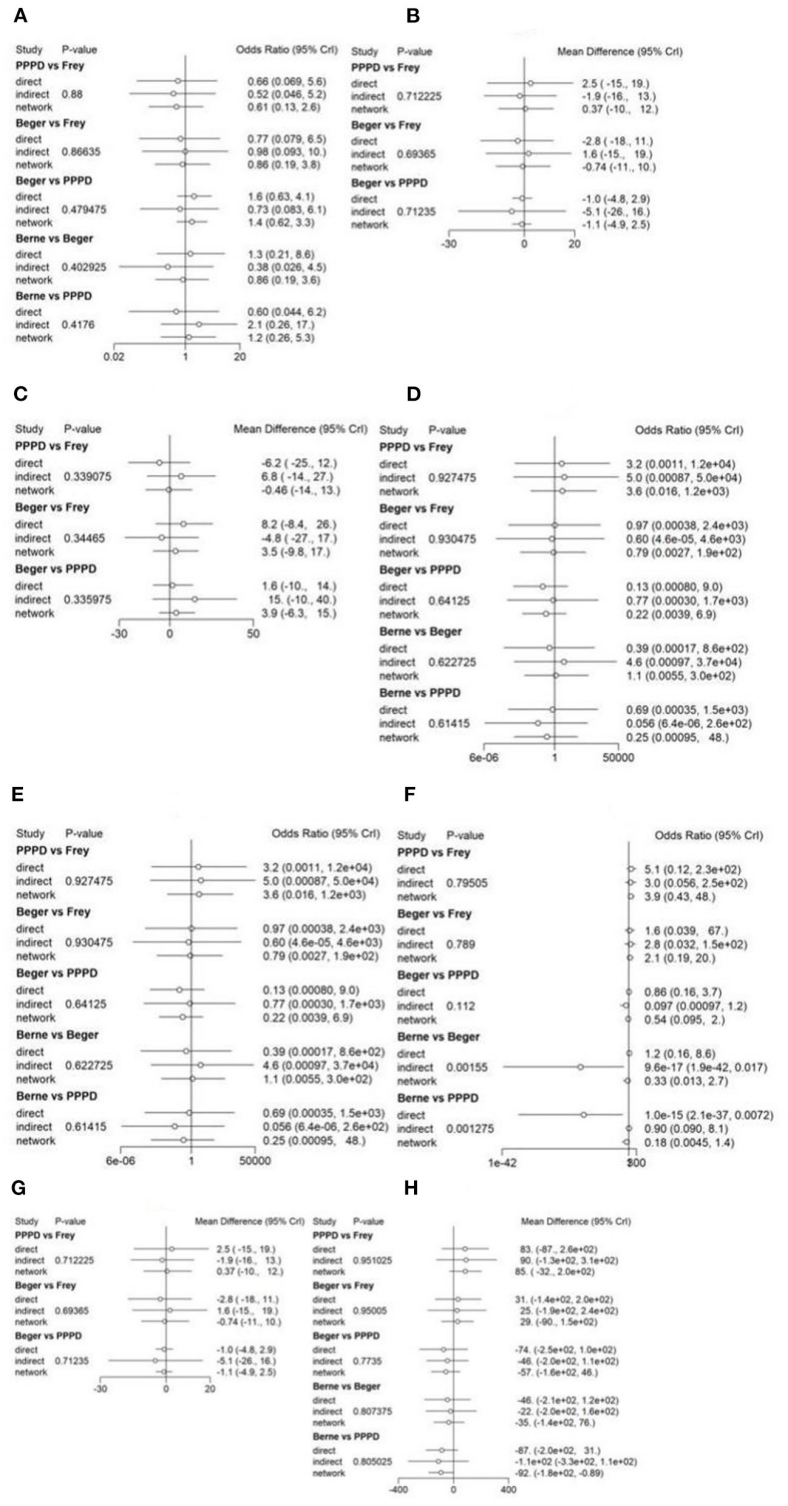
Forest plot of treatment comparisons. The results of continuous variables were estimated as weighted mean difference (WMD) with their corresponding 95% confidence interval (CI), while categorical variables were expressed as odds ratio (OR) with 95% CI. **(A)** Forest plot of short-term pain relief; **(B)** Forest plot of long-term pain relief; **(C)** Forest plot of quality of life; **(D)** Forest plot of pancreatic exocrine function deficiency; **(E)** Forest plot of pancreatic endocrine function deficiency; **(F)** Forest plot of morbidity; **(G)** Forest plot of long-term mortality; **(H)** Forest plot of operation time.

**Figure 4:**
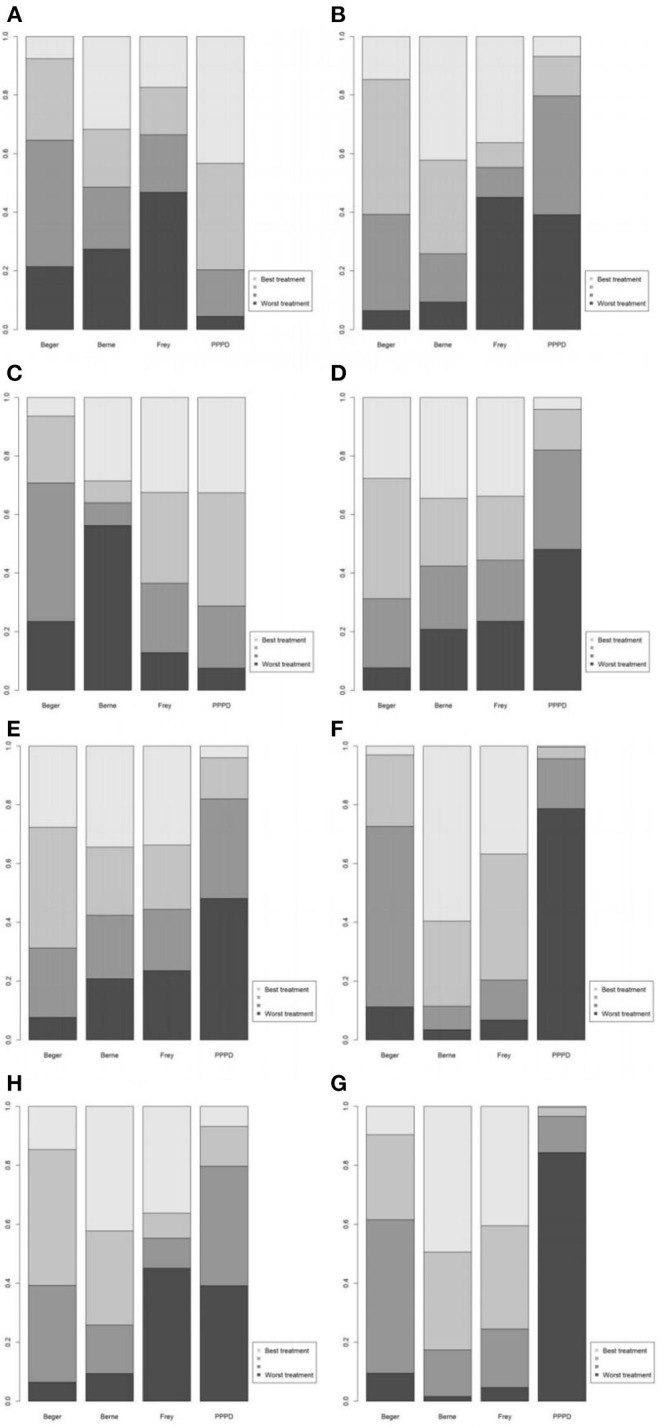
Values of different ranking of the surgical procedures. **(A)** Short-term pain relief; **(B)** long-term pain relief; **(C)** quality of life; **(D)** pancreatic exocrine function deficiency; **(E)** pancreatic endocrine function deficiency; **(F)** morbidity; **(G)** long-term mortality; **(H)** operation time.

Eight studies reported postoperative pain relief data. Three of them reported long-term follow-up results (Figure 2A). The results of NMA for postoperative pain relief are showed in Figure 3A. We found that patients receiving PPPD had significant short-term postoperative pain relief compared to other surgical procedures (PPPD vs. Frey: OR = 0.61, 95% CI 0.13–2.6; Beger vs. PPPD: OR = 1.4, 95%, CI 0.62–3.3, Berne vs. PPPD: OR = 1.2, 95% CI 0.26–5.3). The effect of Beger was similar to Frey (Beger vs. Frey: OR = 0.86, 95% CI 0.19–3.8) and Berne (Berne vs. Beger: OR = 0.86, 95% CI 0.19–3.6). The possibility value of different ranking of each surgical approach is showed in Figure 4A. The highest probability of being ranked first for short-term postoperative pain relief was PPPD, followed by Berne, Frey and Beger.

For Long-term postoperative pain relief (Figure 2B), the NMA results showed that Berne had the highest possibility of being first rank for long-term pain relief and the worst treatment was Frey (Figure 4B). Compared to other treatments, Frey also had the worse long-term pain relief (PPPD vs. Frey: mean difference:0.37, 95% CI −10.0 to 12.0; Beger vs. Frey: mean difference −0.74, 95% CI −11.0 to 10.0; Figure 3B).

### Quality of Life

Four studies reported QOL using the SF-36 QOL instrument (Figure 2C). The NMA results of postoperative QOL are summarized in Figure 3C. We found that PPPD and Frey had the highest probability of being ranked first for QOL (Figure 4C) and both surgical procedures had significantly differences compared to Beger (Beger vs. PPPD: mean difference 3.5, 95% CI −9.8 to 17.0; Beger vs. Frey: mean difference 3.9, 95% CI −6.3 to 15.0).

### Pancreatic Function

Seven studies reported data on pancreatic exocrine function after surgery (Figure 2D) In Figure 3D, PPPD had significant postoperative pancreatic exocrine function deficiency (PPPD vs. Frey: OR 3.6; Beger vs. PPPD: OR 0.22; Berne vs. PPPD: OR 0.25). The effect of Berne is similar to Beger (Berne vs. Beger: OR 1.1). Berne and Frey had a better prognosis in postoperative pancreatic exocrine function deficiency, while PPPD are easier link to worse pancreatic exocrine function (Figure 4D).

Eight studies reported available data about new-onset diabetes after surgery (Figure 2E). The possibility value of the difference rankings of each surgical procedure (Figure 4E) shows the best treatment is Beger and the worst treatment is Frey. In Figure 3E, Beger had the relatively low risk of postoperative pancreatic endocrine function deficiency (Beger vs. PPPD: OR = 0.52 95% CI 0.17–1.3; Beger vs. Frey: OR = 0.36, 95% CI 0.076–1.5; Berne vs. Beger: OR = 1.3, 95% CI 0.26–4.8).

### Morbidity

Eight studies reported available data about morbidity (Figure 2F). The NMA results showed that PPPD had the higher morbidity compared with other treatment (PPPD vs. Frey: OR = 3.9, 95% CI 0.13–48.0; Beger vs. PPPD: OR = 0.54, 95% CI 0.095–2.0; Berne vs. PPPD: OR = 0.18, 95% CI 0.0045-1.40; Figure 3F). In Figure 4F, we found that Berne had the highest possibility value of being ranked first for morbidity, followed by Frey, Beger, and PPPD.

### Long-Term Mortality

Five studies reported more than 10 years of available data about long-term mortality (Figure 2G). In Figure 4G, the ranking of these 4 treatment showed that the best treatment is Berne and the worst treatment is Beger. From the result of NMA, Beger had the significant worse long-term mortality (Beger vs. Frey: mean difference = 0.25, 95% CI −0.52 to 1.1; Beger vs. PPPD: mean difference 0.38, 95% CI −0.34 to 1.0; Figure 3G).

### Operation Time

Six studies reported available data about operation time (Figure 2H). As shown in Figure 3H, the Frey modification was associated with a higher likelihood of the reduced operation time than the Beger procedure, but no significant difference was found between the two sets of procedures Compared with PPPD, the Frey modification required a shorter operation time. In the resection vs. resection group, Beger procedures achieved a shorter operation time than PPPD procedure. The Berne modification tended to have a shorter operation time than the PPPD procedure. The Berne modification also achieved a shorter operation time than the Beger procedure (Figure 4H).

## Discussion

The most common and predominant symptom of CP is pain and the first goal of the treatment of CP is to resolve this intractable pain. To achieve this result, we need to eliminate the cause of the pain, which is now considered to be chronic inflammation. Chronic inflammation induces not only pancreatic neuritis, fibrosis and ductal hypertension (9, 44, 45); ductal hypertension may further exacerbate inflammation (46).

The ideal procedure to treat CP should relieve the pain for a long time, preserve most pancreatic function and provide a high quality of life with a less invasive operation and less trauma (47). Now we have several treatments for CP: medicine, endoscopy and surgery. Medicine and endoscopy are easy to carry out without pancreas damage. However, they cannot offer long-term pain relief (10–12) and cannot reduce the incidence of pancreatic adenocarcinoma when compared with surgery. Therefore, surgery is an effective choice for the treatment of CP. Even early surgery (13–15) may be an option for treating CP.

Many surgical procedures have been developed for CP, such as the Whipple procedure, Beger procedure, Berne modification, Frey modification and so on. All of these surgeries focus on ductal decompression to alleviate pain from obstruction and to prevent inflammatory consequences in the surrounding tissue (48–50). However, the best option for CP remains controversial. Several systematic reviews have been performed to compare the efficacy of these procedures; however, most of them compared just two procedures or included retrospective studies. Here, we compared all of the surgical procedures for CP with only RCT studies. We hope to provide high-quality evidence regarding the best options of surgical procedures for CP.

Our network meta-analysis results showed PPPD had a significantly short-term pain relief and Berne had a higher pain relief effect during long-term follow-up. Besides, the Berne modification also had a relatively better short-term pain relief compared to the Frey modification and Beger procedure.

We also compared QOL, pancreatic exocrine function, new-onset diabetes, long-term mortality and morbidity of CP patients among the surgical procedures. Both PPPD and Frey modification had similar effect on postoperative QOL, but according to our results these two surgical procedures had significant high risk of postoperative pancreatic function deficiency (exocrine function or endocrine function). Compared to other procedures, the Berne modification had relatively low risk of pancreatic function deficiency, but a worse postoperative QOL and lower long-term mortality.

The Beger procedure and PPPD are more complicated surgical procedures than the Frey modification and Berne modification. The Beger procedure and PPPD not only lead to far longer operation times, intensive care monitoring and hospitalization times but also necessitate more frequent blood transfusions (51).

Some studies have reported that patients who underwent PPPD had a high morbidity, i.e., up to 50% (30), and high pancreatic exocrine or/and endocrine dysfunction rates (52). The loss of disease-free neighboring organs is an additional disadvantage of PPPD (30) and frequently leads to dumping complaints and episodes of cholangitis (53). According to our results, although PPPD had a better short-term pain relief, but the effect for long-term pain relief is not good with high risk of pancreatic function deficiency. All of these results indicate that PPPD may not be the best choice for CP with prolonged operation time, raised comorbidity rates and less effect on pain control than organ-preserving surgical procedures.

Based on our results, the Berne modification had a significantly long-term pain relief, relatively low risk of postoperative pancreatic function deficiency and lower long-term mortality. All three of these procedures are organ-preserving surgical procedures. However, the Beger procedure resects the pancreatic head completely; the Frey modification and Berne modification only require a local or subtotal excision of the pancreatic head (30, 54). Therefore, the Frey modification and Berne modification are easier than the Beger procedure. Several studies have reported that the Frey modification requires a shorter operation time and requires less transfused blood units than the Beger procedure (20, 53). One meta-analysis that included one RCT and two non-RCTs reported that the Frey modification had a lower morbidity than the Beger procedure (20). Although there was no difference in the morbidity of the Berne modification and that of the Beger procedure even after the 10-year follow-up (36), surgery could be performed significantly faster with the Berne modification. The total length of hospital stay was also shorter following the Berne modification. These results indicated that the Berne modifications might be safer for patients and had a better effect on pain relief.

There are several limitations to this meta-analysis. First, although only RCTs were enrolled in this study, some of them did not report the methods of randomization and concealment in detail. Then, the main surgical treatments of CP focus on the removal of the mass of the pancreatic head and drainage to relieve the pain and to preserve pancreatic function. Although one study (41) showed a better treatment outcome of distal pancreatectomy with end-to-side pancreaticojejunostomy than PPPD in patients with small pancreatic duct, distal pancreatectomy is not a surgical technique regularly used for the treatment of most CP patients.

## Conclusion

In summary, the main surgical procedures including PPPD, the Beger procedure, the Frey modification and the Berne modification can efficaciously treat CP, not only in the short term but also in the long term. Furthermore, the Berne modification are easier procedures than PPPD and the Beger procedure. However, the Berne modification may lead to poor QOL after surgery. In addition, when CP patients have a mass in the head of the pancreas that cannot be distinguished from pancreatic cancer, based on our experience, PPPD or classical pancreaticoduodenectomy should be the only legitimate choice.

## Data Availability Statement

The raw data supporting the conclusions of this article will be made available by the authors, without undue reservation.

## Author Contributions

N-WK and X-BL designed the study. H-YC and YS acquired the data. WH adjudicated when the consensus of two authors failed. XW analyzed and interpreted the data. YM and YS wrote the paper. N-WK critically revised the manuscript for important intellectual content. All authors contributed to the article and approved the submitted version.

## Funding

This research was supported by Sichuan Province Science and Technology Planning Project (2020YFS0262), West China Hospital Clinical Research Incubation Project (21HXFH058), and the 1.3.5 Project for Disciplines of Excellence-Clinical Research Incubation Project (ZY2017302 and ZYJC21037), West China Hospital, Sichuan University.

## Conflict of Interest

The authors declare that the research was conducted in the absence of any commercial or financial relationships that could be construed as a potential conflict of interest.

## Publisher's Note

All claims expressed in this article are solely those of the authors and do not necessarily represent those of their affiliated organizations, or those of the publisher, the editors and the reviewers. Any product that may be evaluated in this article, or claim that may be made by its manufacturer, is not guaranteed or endorsed by the publisher.

## Abbreviations

CP: chronic pancreatitis
PPPD: pylorus-preserving pancreaticoduodenectomy
QOL: quality of life
SCIE: Science Citation Index Expanded
RCT: randomized controlled trial
CI: confidence interval.
